# Ebstein's Anomaly, Left Ventricular Noncompaction and Gerbode-Like Defect Triad (Fetal Diagnosis and Neonatal Course)

**DOI:** 10.1155/2021/9969588

**Published:** 2021-11-11

**Authors:** Mohammad Mehdi, Snigdha Bhatia, Mehul Patel, Ashraf Aly

**Affiliations:** ^1^Department of Pediatrics, University of Texas Medical Branch, Galveston, TX 77555, USA; ^2^Division of Pediatric Cardiology, University of Texas Health McGovern Medical School, Houston, TX 77030, USA; ^3^Division of Pediatric Cardiology, University of Texas Medical Branch, Galveston, TX 77555, USA

## Abstract

Ebstein's anomaly is characterized by the apical displacement of the septal and posterior leaflets of the tricuspid valve with atrialization of the right ventricle (RV). It is commonly associated with other heart defects including left ventricular noncompaction. We describe a case of prenatally diagnosed Ebstein's anomaly in association with left ventricular noncompaction and a septal defect between the left ventricle and the atrialized portion of the RV (Gerbode-like defect). The patient underwent a modified Blalock−Taussig shunt followed by Glenn procedure because of severe RV hypoplasia and RV outflow tract obstruction. The patient tolerated both procedures and is doing clinically well in anticipation of Fontan procedure for single ventricle palliation.

## 1. Introduction

Ebstein's anomaly (EA) is a rare congenital heart defect with an incidence of 0.5 in 100,000 live births [1] and was first described in 1866. Its predominant features are adherence of the septal and posterior leaflets of the tricuspid valve to the underlying right ventricular (RV) myocardium, apical displacement of the functional tricuspid valve, and atrialization of the RV. EA is primarily a right-sided heart defect, and recent literature shows that it can be associated with left-sided heart lesions in up to 39% of cases [2]. The hemodynamics in EA is dictated by the distortion of the anterior leaflet of the tricuspid valve and the degree of atrialization of the RV [2].

Left ventricular noncompaction (LVNC) is the third most diagnosed cardiomyopathy with an estimated prevalence of isolated LVNC ranging from 0.05 to 0.14 in adults [3, 4]. It is characterized by prominent trabeculae, intratrabecular recesses, and two distinctive layers in the myocardium: compacted and noncompacted [5, 6]. LVNC-like morphology is seen in greater than 18% patients with EA [5]. Recent literature has shown *β*-myosin heavy chain 7 (MYH7) mutation and *α*-tropomyosin 1 (TPM1) gene mutations to be associated with EA-LVNC [6, 7]. The cumulative effect of EA and LVNC in affecting ventricular function is not well known.

Gerbode defect is defined as an abnormal shunting between the LV and the RA. Gerbode-like defect is shunting between the LV and the atrialized portion of the RV in case of EA. While EA has been associated with septal defects, there have only been two reports of its occurrence with a Gerbode-like defect and neither of the two cases reported left-sided cardiac defects [8, 9]. To our knowledge, this is the first reported case of a Gerbode-like defect in a patient with EA-LVNC.

## 2. Case Presentation

**Figure 1:**
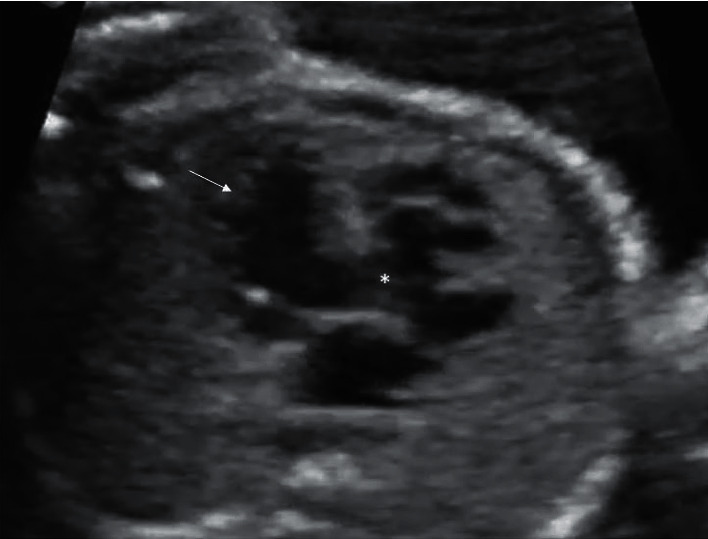
A 4-chamber view in a fetal echocardiogram showing a heavily trabeculated spongy looking LV suggestive of non-compaction (arrow). It also shows the Ebstein's anomaly and a VSD (^*∗*^) between the LV to the atrialized portion of the RV.

**Figure 2:**
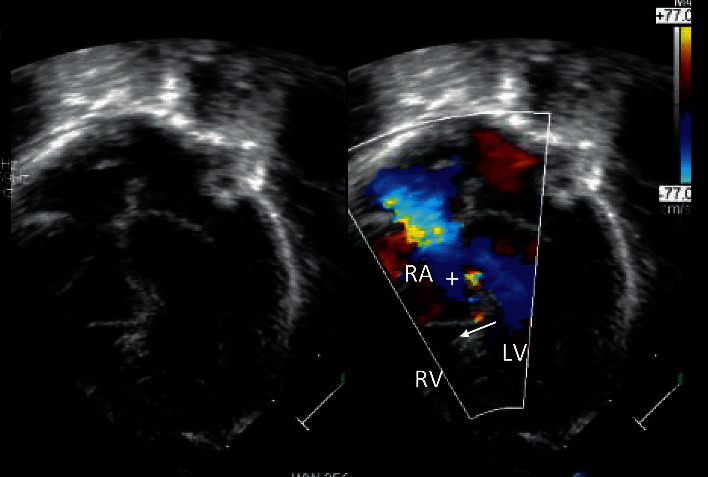
Transthoracic echocardiogram with 2D and color Doppler showing an apical displacement of septal leaflet of the tricuspid valve (arrow) and atrialization of RV. A left to right shunt is also seen across a VSD into the atrialized RV.

**Figure 3:**
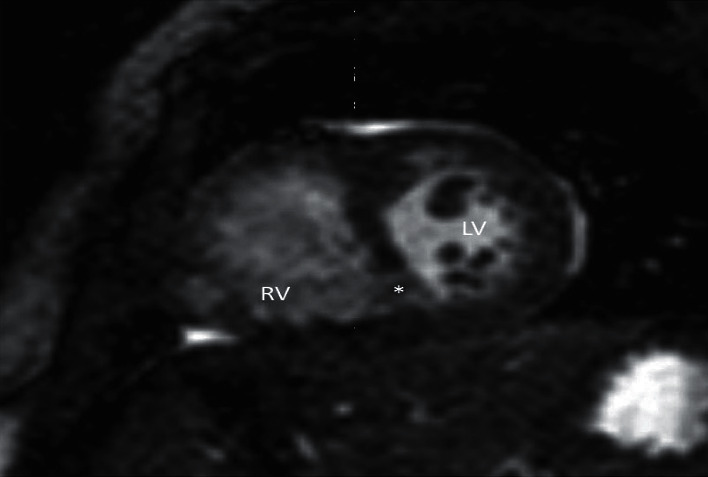
Short axis cine SSFP image of hypoplastic RV, VSD (^*∗*^) and normal sized LV. Note the abnormal trabeculations along the lateral and apical LV walls.

A 17-year-old primigravida was referred to the fetal cardiology clinic for a suspected congenital heart defect. The fetal echocardiogram (ECHO) was significant for EA, hypoplastic RV, a septal defect between the left ventricle and the atrialized portion of the RV (Gerbode-like defect) in addition to possible LVNC (Figure 1). These findings were confirmed by postnatal echocardiography which also showed a moderate to severe degree of RV outflow tract obstruction, multiple small ventricular septal defects, and left ventricular noncompaction (Figure 2). Chromosomal microarray was remarkable for a gain chromosome band 15q11.2 and 1q44. A panel of 17 genes was tested, which included TPM1 and MYH7, and were all negative. The neonate did well initially, but his oxygen saturation decreased gradually as his RV outflow tract obstruction progressed. Cardiac MR was performed at 5 months of age, prior to surgical intervention, which showed severe dilatation of the right atrium and an atrialized right ventricle. As seen in Figure 3, the RV was severely hypoplastic, with an RV indexed end diastolic volume of 32 ml/m^2^ and normal systolic function with an ejection fraction of 58%. The left ventricle was of normal size with an indexed end diastolic volume of 52 cc/m^2^ and normal systolic function with an ejection fraction of 73%. There was excessive trabeculation of the left ventricle with a ratio of noncompact to compact myocardium of approximately 4 : 1. Late gadolinium enhancement imaging was not performed.

At 7 months of age, he underwent cardiac catheterization which showed severe EA, multilevel right ventricular outflow tract (RVOT) obstructions, Swiss cheese ventricular septum, Gerbode-like defect, and a moderate size secundum ASD. There was a minimal forward flow through the RVOT, and the Qp : Qs ratio was 0.3 : 1. A Blalock–Taussig shunt was placed at that time.

**Figure 4:**
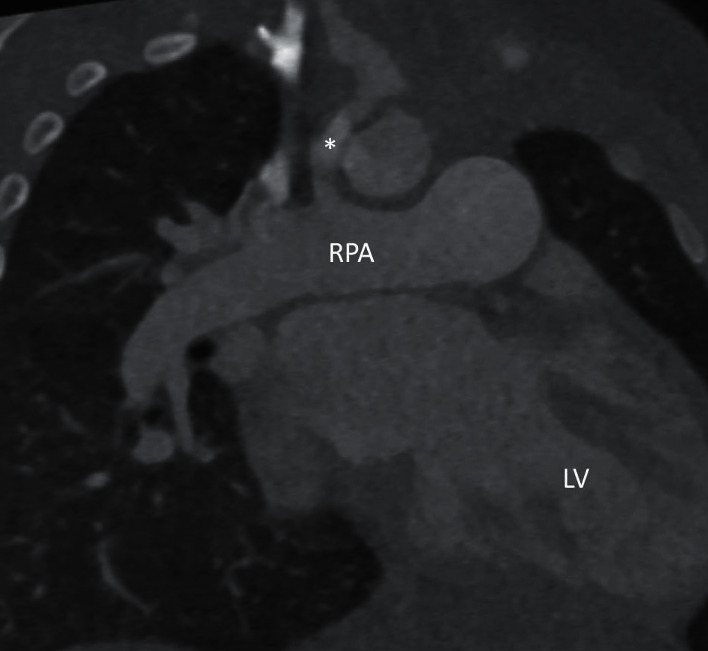
An oblique coronal image from contrast enhanced cardiac CT showing a right modified Blalock-Taussig shunt (^*∗*^), dilated right pulmonary artery (RPA), left atrium, and LV. Note the abnormal LV trabeculations along the LV inferior, lateral and apical walls.

**Figure 5:**
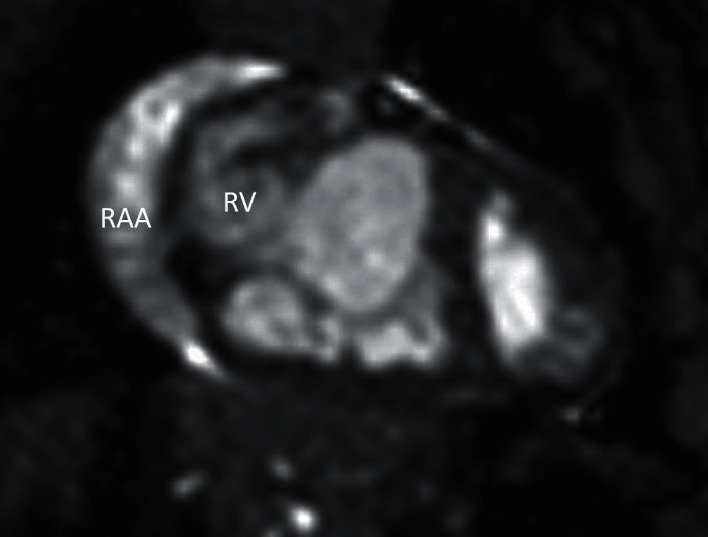
A coronal cine SSFP image from cardiac magnetic resonance of the trabecular, hypoplastic RV and the large atrialized portion of the RV. The dilated right atrial appendage (RAA) is also seen.

Subsequent follow-up imaging studies were done at 14 months of age and included cardiac magnetic resonance imaging and cardiac computerized tomography (CT) scan which showed a severely hypoplastic RV with severe multilevel RVOT stenoses. The severely dilated right atrium with an atrialized right ventricle was also redemonstrated (Figures 4 and 5). Interestingly, the cardiac CT angiogram showed that the right pulmonary artery was diffusely dilated, measuring 15.3 × 13.4 mm, and was likely related to the flow through the right BT shunt. The main pulmonary artery was mildly hypoplastic, and the left pulmonary artery was of normal size. Hemodynamic findings during cardiac catheterization at that time showed a Qp/Qs of 0.95.

At 15 months of age, he underwent Glenn procedure. At his most recent cardiac evaluation, the O2 saturation was 92% on room air, and his Glenn shunt continues to exhibit a laminar flow at low velocity. The decision to proceed with a single ventricle palliation was based on the fact that the RV is severely hypoplastic and would not be able to support the pulmonary circulation in addition to the severe multilevel RVOT obstruction.

## 3. Discussion

LVNC has multiple known associations such as arrhythmias, congenital heart disease such as septal defects, right heart obstructive lesions, hypoplastic left heart syndrome, and EA [10]. In a study of patients with LVNC, the prevalence of EA was highest (15%), followed by aortic coarctation (3%), tetralogy of Fallot (2%) and uni- or bicuspid aortic valves (1%) [11]. It was hypothesized that genetic abnormalities and fetal hemodynamics may result in structural defects and impaired LV myocardial development. The genes are most associated with EA-LVNC code for sarcomeric proteins, which suggests that a similar genetic predisposition may lead to defective right and left ventricular myocardial differentiation with different morphologic-phenotypic manifestations.

The first three cases of the EA-LVNC association were reported in 2004; since then, there have been several reports describing the association [12]. Recent literature has shown two main genetic associations; MYH7 and TPM1 gene mutation [6, 7], both of which were negative in our patient.

Patients with EA-LVNC may present with a wide range of symptoms such as right-sided heart failure, cyanosis, arrhythmia, and sudden cardiac death. Studies in younger children showed a worse outcome in patients with EA and LVNC compared to those with EA alone [13]. A preterm infant in Qatar was the youngest patient to be diagnosed with combined LVNC and EA after presenting with neonatal cardiac failure [14]. Our patient was diagnosed prenatally by a fetal ECHO, which was confirmed postnatally.

A left ventricle to right atrial shunt is rare and accounts for <1% of congenital cardiac defects [15]. The downward displacement of the tricuspid valve leaflets may contribute to the development of a LV to the atrialized RV shunt through the VSD called a Gerbode-like defect. It has been hypothesized that the right atrial volume overload caused by the left to right shunt can result in reversal of shunt and development of cyanosis. In the case of our patient, this was further exacerbated by the RVOT obstruction [16]. Although septal defects had been documented in patients with EA, only two cases have been reported of Gerbode-like defects, and neither of these cases reported left ventricular noncompaction [11, 12].

To our knowledge, our patient is the first reported case of an EA- LVNC- Gerbode-like defect triad. While expected genetic abnormalities associated with EA-LVNC such as TPM1 and MYH7 were negative, there may be an undiagnosed genetic association with these concurrent defects. Early diagnosis of these defects may contribute to better management and outcomes.

## Data Availability

Data used to support the findings of this study are available from the corresponding author upon request.
